# The Association Between Gene Polymorphisms and Leukocytosis with Thrombotic Complications in Patients with Essential Thrombocythemia and Polycythemia Vera

**DOI:** 10.5505/tjh.2012.03780

**Published:** 2012-06-15

**Authors:** Özgür Mehtap, Elif Birtaş Ateşoğlu, Pınar Tarkun, Emel Gönüllü, Hakan Keski, Yıldıray Topçu, Nilüfer Üzülmez, Deniz Sünnetçi, Abdullah Hacıhanefioğlu

**Affiliations:** 1 Kocaeli University, School of Medicine, Department of Hematology, Kocaeli, Turkey; 2 Kocaeli University, School of Medicine, Department of Internal Medicine, Kocaeli, Turkey; 3 Kocaeli University, School of Medicine, Department of Medical Genetic, Kocaeli, Turkey

**Keywords:** PAI-1 4G/5G, ACE I/D, Polycythemia vera, Essential thrombocythemia, Thrombosis, Leukocytosis

## Abstract

**Objective: **Vascular events are a common complication in patients with polycythemia vera (PV) and essential thrombocythemia (ET). This study aimed to analyze the association between PAI-1 4G/5G and ACE I/D gene polymorphisms, and leukocytosis with thrombosis in patients with PV and ET.

**Material and Methods:** In total, 64 patients with ET and PV were evaluated. Arterial or venous thrombosis, such as cerebral transient ischemic attack, ischemic stroke, myocardial infarction, peripheral arterial thrombosis, deep venous thrombosis, and pulmonary embolism, were defined as a vascular event. DNA samples were screened for mutations via reverse hybridization strip assay.

**Results:** In terms of PAI-1 gene polymorphism, the frequency of the 4G and 5G allele was 48.5% and 51.5%, respectively. The ACE allele frequency was 51.2% and 48.8% for D and I, respectively. There wasn’t an association between occurrence of vascular events and the frequency of any allele. In terms of occurrence of vascular events, there weren’t any significance differences between the patients that were carrying the ACE D/D homozygous allele to ACE I/D and those that carried the I/I allele (P = 0.93). There wasn’t a significant difference in occurrence of vascular events between the PAI-1 5G/5G homozygote allele carriers, and the 4G/5G and 4G/4G allele carriers (P = 0.97). Vascular events were significantly more common in the patients with leukocytosis (leukocyte count >10 × 109 L–1) than in those without leukocytosis (leukocyte count ≤10 × 109 L–1) (P = 0.00). Age >60 years was also a significant risk factor for occurrence of vascular events(P = 0.008).

**Conclusion:** PAI-1 and ACE gene polymorphisms were not considered new risk factors for thrombosis in PV and ET patients. On the other hand, leukocytosis at diagnosis was associated with the occurrence of vascular events in the patients with ET and PV.

## Abstract (tr)

**Amaç:** Polisitemia vera (PV) ve esansiyel trombositemi (ET)’de vasküler olaylar sık görülen komplikasyonlardır. Bu çalışmada, PV, ET hastalarında PAI-1 4G/5G, ACE I/D gen polimorfizmi ile lökositoz ve bunların vasküler olaylarla ilişkisini araştırmayı amaçladık.

**Gereç ve Yöntemler:** PV ve ET’li 64 hasta çalışmaya alındı. Serebral geçici iskemik atak, iskemik inme, miyokard infarktüsü, periferik arteryel tromboz, derin ven trombozu ve pulmoner emboli gibi arteryel veya venöz trombozlar vasküler olay olarak tanımlanmıştır. DNA örnekleri mutasyonlar yönünden ters hibridizasyon strip testi ile araştırıldı.

**Bulgular:** PAI-1 gen polimorfizmine bakıldığında 4G ve 5G allel sıklığı sırası ile %48,5 ve %52,5 tespit edildi. ACE geninin D ve I sıklıkları sırası ile %51,2 ve %48,8 bulundu. Vasküler olaylar ile allel sıklıkları arasında anlamlı ilişki saptamadık. Vasküler olaylar değerlendirildiğinde, ACE D/D homozigot allel taşıyan hastalar ile ACE I/D ve I/I allel taşıyanlar arasında anlamlı fark tespit edemedik (P = 0,93). PAI-1 5G/5G homozigot allel taşıyıcıları ile 4G/5G ve 4G/4G allel taşıyıcıları karşılaştırıldığında vasküler olay görülmesi açısından anlamlı farklılık tespit edilmedi (P = 0,97).

Vasküler olayın, lökositozu (>10x10^9^/L) olan hastalarda, lökositozu olmayanlara göre belirgin olarak daha sık görüldüğü saptandı (P = 0,00). Ayrıca tanı anındaki lökositoz varlığının vasküler olay riskini belirgin olarak arttırdığı tespit edildi (OR, 15,625[95% CI, 4,322 -56,492]). Yaşın altmıştan fazla olması vasküler olay için diğer risk faktörü olarak saptandı (P = 0,008)

**Sonuç:** PAI-1 ve ACE gen polimorfizmi, PV ve ET hastalarında tromboz için ek bir risk faktörü olarak kabul edilemez. Ancak, tanı sırasında lökositoz vasküler olaylar için risk faktörüdür.

## INTRODUCTION

Thrombotic events, both in the arterial and venous systems, are a common complication in patients with polycythemia vera (PV) and essential thrombocythemia (ET). About 30%-50% of patients with PV and ET suffer from these complications, and vascular mortality accounts for 35%-45% of all deaths [1,2,3]. Studies report that age is among the most important risk factors for thrombosis [3]. While some researchers suggest that a previous thrombotic complication is another independent and predictive factor for recurrent thrombosis, others emphasize the association between JAK2V617F mutation and thrombotic complications [4,5]. Landolfi and Carobbio reported that leukocytosis was a risk factor for thrombosis in patients with PV and ET [6,7]. 

The formation of thrombus depends on the balance between fibrinolytic and procoagulant systems. During fibrinolysis plasminogen is converted to plasmin, which in turn dissolves fibrin clots. This conversion is regulated by tissue plasminogen activator and plasminogen activator inhibitor-1 (PAI-1)—a serine protease inhibitor [8]. The PAI-1 gene has polymorphisms in the promoter region, in which 1 allele has a 4-guanosine sequence [4G) and the other has a 5-guanosine sequence (5G) [9]. The PAI-1 4G and 5G alleles both have a binding site for an activator of transcription; however, the 5G allele has an additional binding site for a repressor, which leads to lower transcription rates and less PAI-1 activity than 4G allele[10]. As such, the 4G allele leads to moderately higher plasma PAI-1 levels than 5G allele and may promote pathological fibrin deposition and thrombotic events [11,12]. 

Rigat et al. (1990) reported a polymorphism involving the presence (insertion, I) or absence (deletion, D) of a 287-bp sequence of DNA in intron 16 of the angiotensin I-converting enzyme (ACE) gene. According to this polymorphism, 3 genotypes (I/I, I/D, and D/D) were determined. The D/D genotype has been associated with the highest plasma ACE activity, the I/D genotype with moderate ACE activity, and the I/I genotype with the low ACE activity [13]. It has been reported that individuals with the D allele—especially the DD genotype—are prone to hypertension [14,15,16], acute myocardial infarction (AMI) [17], and ischemic stroke [18,19]. 

To date, no study on PAI-1 4G/5G and ACE I/D gene polymorphism in relation to the risk of thrombosis in patients with PV and ET has been published. As such, using our patient database the present study aimed to analyze the association between these polymorphisms, and age and leukocytosis with thrombosis in patents with PV and ET.

## MATERIALS AND METHODS

**Patients**

This retrospective cohort study included patients with ET and PV that were diagnosed according to WHO-2008 criteria [20]. All analyses were performed using data for 64 patients with ET and PV that were followed-up at Kocaeli University, School of Medicine, Department of Hematology, between 2005 and 2010. We collected data regarding laboratory values, and clinical features at diagnosis, and every 3 months during follow-up. Arterial or venous thrombosis, such as cerebral transient ischemic attack (TIA), ischemic stroke, AMI, peripheral arterial thrombosis (PAT), deep venous thrombosis (DVT), and pulmonary embolism (PE), were defined as a vascular event. The events of interest were thromboses that occurred as the initial manifestation of disease or during follow-up. Initial thromboses were events that occurred up to 2 years prior to diagnosis, based on the fact that 75% of thromboses indicative of PV occur during this time period [1]. 

Cerebral computed tomography (CT) or magnetic resonance imaging (MRI) was used to diagnose ischemic stroke and neurologic symptoms. In addition to these procedures, electrocardiography and/or increased cardiac enzymes, angiography, ultrasonography of the arms or legs, and pulmonary ventilation-perfusion scan and/ or CT were used to diagnosis TIA, AMI, PAT, DVT, and PE, respectively. Microcirculatory events, such as vascular headaches, dizziness, distal paresthesia, etc., were not considered vascular events and were not analyzed. The study was conducted in accordance with good clinical and laboratory practices, and the principles of the Declaration of Helsinki. The University of Kocaeli Ethics Committee approved the study protocol. 

**Genotyping**

**DNA extraction**

Genomic DNA was extracted from 3 μL of peripheral blood with EDTA using the MagNA Pure Compact Nucleic Acid Isolation Kit I (Roche. Diagnostics GmbH, Germany) and was stored at 4 °C until analyzed. Quantification of the DNA concentration was performed using a NanoDrop ND-1000 spectrophotometer. The ratio of spectrophotometric measurements at 260 nm and 280 nm provided an estimate of the purity of the DNA sample. 

**Multiplex PCR-CVD StripAssay **

Isolated DNA samples were screened for mutations associated with cardiovascular disease using reverse hybridization strip assay (Vienna Lab, CVD StripAssay, GMBH, Austria). This assay covers PAI-1, 4G/5G, and ACE I/D mutations. For each reaction 5 μL of DNA was added to 20 μL of PCR reaction mix containing 15 μL of amplification mix and 5 μL of diluted Taq DNA polymerase (1U). PCR was performed in 0.2-mL tubes under the following cycling conditions: initial denaturation at 94 °C for 2 min, 35 cycles at 94 °C for 15 s, 58 °C for 30 s, 72 °C for 30 s, and a final extension step at 72 °C for 3 min. To confirm amplification, each PCR product was electrophoresed on 3% agarose gel. Finally, the amplification products were selectively hybridized to a test strip that contained allelespecific (wild type and mutant) oligonucleotide probes immobilized as an array of parallel lines. 

Bound biotinylated sequences were detected using streptavidin-alkaline phosphatase and color substrates. Hybridization was performed in an automated incubator (profiBlot T48, TECAN, Männedorf, Switzerland). For each polymorphic position, 1 of 3 possible staining patterns may be obtained: normal genotype (wild type probe only), heterozygous genotype (wild type and mutant probe), and homozygous mutant genotype (mutant probe only). 

**Mutation detection using real-time PCR **

JAK2 gene mutations were detected using a LightCycler 1.5 (Roche Diagnostics, Mannheim, Germany) and JAK2V617F LightMix Kit (TIB MOLBIOL, GmbH, Berlin, Germany), and capillary tubes. According to the manufacturer’s protocol, 20 uL of reaction containing 5 uL of genomic DNA and 15 uL of reaction mix (PCR-grade water, 25 mM Mg+2 solution, primer, probe, and enzyme mix) was prepared. Each study was carried out with negative control (5 uL of PCR-grade water) and positive controls that were available in commercial kits. Data obtained were analyzed using LightCycler v.3.5 software. Mutations were evaluated according to the status of the normal allele or the mutant, in consideration of the specific melting points (Tm). 

**Statistical analysis**

All analyses were performed using SPSS v.15.0 for Windows XP (SPSS, Chicago, IL, USA). Pearson’s chisquare and Fisher’s exact test were used to compare categorical variables. The Mann-Whitney U test was used to measure the statistical significance of the leukocyte count at diagnosis and its association with occurrence of vascular events. The relationship between vascular events and an above normal leukocyte count was evaluated using Pearson’s chi-square test. The frequency of alleles and their significance were determined using the Mann-Whitney U test. 

P values <0.05 were considered statistically significant. 

## RESULTS

In total, 64 patients were evaluated. Patient characteristics are summarized in Tables 1 and Table 2. JAK2 mutation positivity did not differ significantly between the patients with and without vascular events (P = 0.374 [Pearson’s chi-square analysis]). In terms of PAI-1 gene polymorphism, the frequency of the 4G and 5G allele was 48.5% and 51.5%, respectively. The ACE allele frequency was 51.2% and 48.8%, respectively, for D and I. There wasn’t an association between occurrence of vascular events and any allele frequency. There wasn’t a significant difference in occurrence of vascular events between the patients that were carrying the ACE D/D homozygous allele to ACE I/D heterozygoues, and I/I homozygote allele carriers (P = 0.93) (OR = 4.67 [95% CI: 0.921-23.710]). Analysis of PAI-1 gene polymorphisms according to vascular events showed that PAI-1 5G/5G homozygote allele carriers did not differ significantly from 4G/5G heterozygote and 4G/4G homozygote allele carriers (P=0.97) (OR=4.00 [95% CI:0.777-20.595]). 

The mean leukocyte count at diagnosis in patients with and without vascular events was 13.4x10[u]9[/u] L[u]–1[/u] ± 3.9x10[u]9[/u] L[u]–1[/u] and 9.8 x 10[u]9[/u] L[u]–1[/u] ± 3.8x x 10[u]9[/u] L[u]–1[/u], respectively. Significantly more vascular events were observed in the patients with leukocytosis (leukocyte count >10 x 10[u]9[/u] L[u]–1[/u]) (P = 0.00) than in those without leukocytosis (leukocyte count ≤10 x 10[u]9[/u] L[u]–1[/u]) and the presence of leukocytosis at diagnosis was a significant risk factor for vascular events (OR = 15.625 [95% CI: 4.322-56.492]). In addition to leukocytosis, age >60 years was a significant risk factor for vascular events (P = 0.008) (OR = 4.154 [95% CI: 1.416- 12.184]) (Table 3).

## DISCUSSION

Thrombosis in patients with PV and ET occurs in arterial, venous, or microcirculatory systems. In the present study the majority of vascular complications were arterial, which is in accordance with previous reports [21]. Age above sixty years was reported to be a major risk factor for vascular complications in patients with ET and PV [3]. In accordance with the literature, patients in the present study aged >60 years had a significantly higher vascular event. 

Hereditary thrombophilic states in patients with PV and ET, including factor V Leiden, prothrombin G20210A, and methylenetetrahydrofolate reductase (MTHFR) mutations, have been studied extensively [22,23); however, other polymorphisms, including PAI-1 4G/5G and ACE I/D gene mutations, haven’t been investigated in patients with PV and ET. It has been suggested that elevated plasma PAI-1 levels associated with the PAI-1 4G allele lead to a hypofibrinolytic state, which could be considered a risk factor for arterial and venous thromboses [11,12]. Data regarding the relationship between PAI-1 polymorphism and the risk for both arterial and venous thrombosis are inconsistent. Some researchers think that the PAI-1 4G allele confers an increase in the risk for stroke and myocardial infarction [24,25,26]. In contrast, other researchers think that the PAI-1 4G/4G genotype does not confer a risk and may even protect against cerebrovascular events [27,28,29,30]. 

Data regarding the association between the presence of the 4G allele and the risk for venous thrombotic episodes are also controversial. A recent meta-analysis reported an unexpected weak association between the 4G allele and the risk of venous thromboembolism, based on analysis of patients with venous thrombosis that were not included in certain risk groups with other genetic or acquired thrombophilic defects [31]. Nonetheless, this polymorphism was reported to increase the risk for venous thrombotic episodes in patients with other congenital or acquired prothrombotic disorders [32,33,34,35]. Although PAI-1 levels weren’t measured in the present study, Cancelas et al. reported that the PAI-1 plasma concentration was significantly higher in patients with ET and PV than in the control group [36]. In the present study there wasn’t a correlation between PAI-1 gene polymorphism (neither 4G/4G and 5G/5G homozygosity nor 4G and 5G allele frequencies) and occurrence of vascular events in patients with PV and ET. 

The correlation between ACE polymorphism and vascular events, especially arterial events, has been studied. Dilley et al. (1998) reported that patients with ACE D/D polymorphism had moderately higher risk for venous thrombosis risk than those with other genotypes (OR = 1.5 [95% CI: 0.9-2.6]). In addition, females with the ACE D/D genotype did not a have an increased risk (OR = 0.9 [95% CI: 0.5-1.9}), whereas males with the genotype had a nearly 3-fold higher risk (OR = 2.8 [95% CI: 1.2- 6.2]) [37]. Cambien et al. reported that there was a positive association between the ACE D allele and myocardial infarction [17]. Their study included 610 patients and 733 controls, and they observed that the ACE DD genotype occurred significantly more frequently in the male patients with myocardial infarction than in the controls [17]. Their results, however, were not replicated in a large study by Agerholm-Larsen et al. that investigated the association between the ACE polymorphism and ischemic heart disease in a case-referent format (n = 10,150) and in a retrospective cohort format (n = 7263); no significant differences in the incidence of myocardial infarction or any other manifestation of ischemic heart disease between the genotype classes were noted [38]. 

Sayed-Tabatabaei et al. suggested that the D allele is not clinically important in the general population, but may play an important role in certain groups of patients with coronary heart disease [39]. While 2 meta-analyses reported a significant positive correlation between the D allele and ischemic stroke, [18,19], the finding was not confirmed by Zee et al. in a subsequent prospective matched case-control study [40]. The present study investigated the relationship between the ACE polymorphism and the occurrence of vascular events, and a statistically significant difference in genotype and allele frequency for ACE polymorphisms was not observed between the patients with and without vascular events. 

In recent years new data concerning the pathogenesis of thrombosis in Philadelphia-chromosome negative myeloproliferative disorders have become available, including the role of leukocyte activation, and leukocyte interaction with platelets and endothelial cells. In myeloproliferative disorders neutrophils circulate in an activated state and are able to bind to platelets in a dynamic adhesive process [41,42,43,44]. This process leads to expression of tissue factors that contribute to endothelial activation and damage [45]. Published data indicate that leukocytosis a novel, powerful risk factor for thrombosis in both PV and ET [6,7]. In the present study the white blood cell count (WBC) at diagnosis was evaluated as well as its association with thrombosis. We observed that WBC levels above normal (>10 x 10[u]9[/u] L[u]–1[/u]) at diagnosis were associated with vascular events in the patients with ET and PV. Landolfi et al. studied patients with PV and reported that the risk of thrombosis was clearly higher in patients with a WBC >10 x 109 L[u]–1[/u] and significantly higher in patients with a WBC >15 x 10[u]9[/u] L[u]–1[/u]. In addition, they reported that leukocytosis was more strongly associated with arterial thrombosis than with venous thromboembolism [6]. Carobbio et al. examined the association between leukocytosis and thrombosis in patients with ET and reported the following: 1. Multi-variable analysis demonstrated that patients with a baseline WBC above the median had a risk of developing thrombosis approximately 2-fold greater than that in patients with a WBC below the median; 2. Cytoreductive therapy lowered the WBC during follow-up, which was associated with a reduction in its thrombogenic effect; 3. The role of the WBC in predicting thrombosis was more evident in untreated low-risk patients than in treated highrisk patients [7]. As previously mentioned, patients in the present study with vascular events had significantly higher leukocyte counts than those without a occurrence of vascular events. 

The research-proven risk factors for thrombosis in patients with ET and PV are age >60 years and a history of thrombosis. The presence of either risk factor places patients in the high-risk category, indicating the need for myelosuppressive therapy [46]. In the present study we expected to find that ACE and PAI-1 gene polymorphisms would be identified as new risk factors for thrombosis; however, the findings did not support our expectations, which may have been due to the small number of patients included. On the other hand, the present findings do support the notion that an elevated WBC at diagnosis could be associated with an increased risk of vascular events in patients with ET and PV. Physicians should be aware of the potential for vascular events in patients with ET and PV that have leukocytosis. As additional data from similar studies accrue over time, patients with leukocytosis could eventually be defined as a high-risk group. In the future patients with leukocytosis might be routinely considered for cytoreductive therapy, especially those aged >60 years and those with a history of thrombosis. 

**Conflict of Interest Statement **

The authors of this paper have no conflicts of interest, including specific financial interests, relationships, and/ or affiliations relevant to the subject matter or materials included.

## Figures and Tables

**Table 1:**
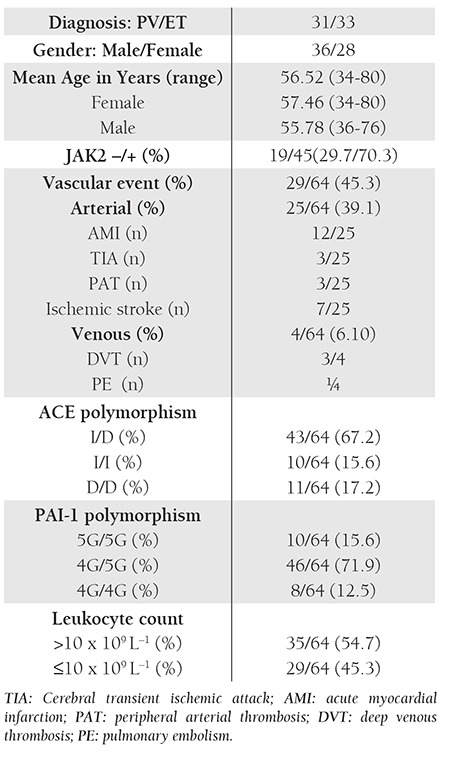
Patient characteristics.

**Table 2:**
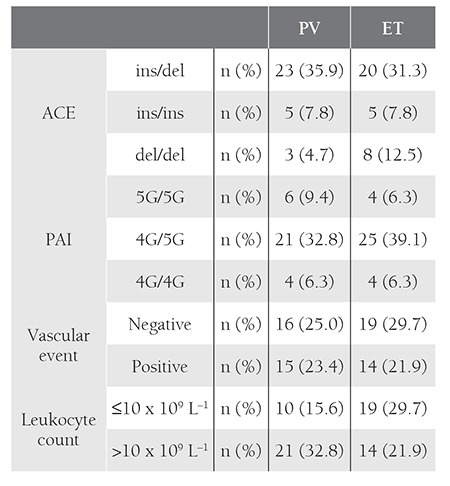
Patient characteristics, according to PV and ET.

**Table 3:**
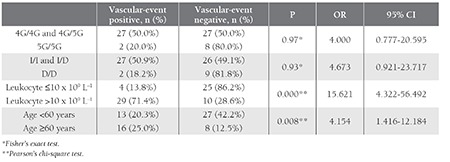
Vascular events in patients
